# Palmitoylation Transduces the Regulation of Epidermal Growth Factor to Organic Anion Transporter 3

**DOI:** 10.3390/pharmaceutics17070825

**Published:** 2025-06-25

**Authors:** Zhou Yu, Jinghui Zhang, Jiaxu Feng, Guofeng You

**Affiliations:** Department of Pharmaceutics, Rutgers, The State University of New Jersey, Piscataway, NJ 08854, USA

**Keywords:** transporter, organic anion transporter 3, epidermal growth factor, protein kinase A, palmitoylation, regulation, signaling pathway

## Abstract

**Background:** Organic anion transporter 3 (OAT3) in the kidney proximal tubule cells plays a critical role in renal clearance of numerous endogenous metabolites and exogenous drugs and toxins. In this study, we discovered that epidermal growth factor (EGF) regulates the expression and activity of OAT3 through palmitoylation, a novel mechanism that has never been described in the OAT field. **Methods/Results:** Our results showed that treatment of OAT3-expressing cells with EGF led to a ~40% increase in OAT3 expression and OAT3-mediated transport of estrone sulfate, a prototypical substrate for OAT3. EGF-stimulated OAT3 transport activity was abrogated by H-89, a protein kinase A (PKA) inhibitor, indicating that an EGF-PKA signaling pathway is involved in the regulation of OAT3. We also showed that treatment of OAT3-expressing cells with EGF resulted in an enhancement of OAT3 palmitoylation, a novel type of post-translational modification for OATs, and such an enhancement was blocked by H-89, suggesting that the EGF-PKA signaling pathway participated in the modulation of OAT3 palmitoylation. Palmitoylation was catalyzed by a group of palmitoyltransfereases, and we showed that OAT3 palmitoylation and expression were inhibited by 2-BP, a general inhibitor for palmitoyltransfereases. We also explored the relationship among EGF/PKA signaling, OAT palmitoylation, and OAT transport activity. We treated OAT3-expressing cells with EGF or Bt2-cAMP, a PKA activator, in the presence and absence of 2-BP, followed by the measurement of OAT3-mediated transport of estrone sulfate. We showed that both EGF- and Bt2-cAMP-stimulated OAT3 transport activity were abolished by 2-BP, suggesting that palmitoylation mediates the regulation of EGF/PKA on OAT3. Finally, we showed that osimertinib, an anti-cancer drug/EGFR inhibitor, blocked EGF-stimulated OAT3 transport activity. **Conclusions:** In summary, we provided the first evidence that palmitoylation transduces the EGF/PKA signaling pathway to the modulation of OAT3 expression and function. Our study also provided an important implication that during comorbidity therapies, EGFR inhibitor drugs could potentially decrease the transport activity of renal OAT3, which would subsequently alter the therapeutic efficacy and toxicity of many co-medications that are OAT3 substrates.

## 1. Introduction

The organic anion transporter (OAT) family is crucial for the movement of organic anions across cell membranes in multiple organs. Organic anion transporter 3 (OAT3), expressed in the kidney proximal tubule cells, plays a critical role in renal clearance of a wide range of substances, including endogenous metabolites and exogenous drugs and toxins [1,2,3,4,5]. For instance, drug substrates of OAT3 could have altered pharmacokinetic profiles in individuals with impaired OAT3 function or patients on medications that inhibit OAT3 function, causing unexpected toxicity. Given its importance in drug elimination and toxicity, OAT3 is a critical target for the pharmaceutical industry, especially in drug design and development [3,4,6,7]. Therefore, understanding the regulation of OATs can shed light on their complex roles in renal diseases and develop potential therapies for conditions like chronic kidney disease or drug-induced nephrotoxicity. Thus, research into OATs is of great clinical importance for ensuring safe and effective drug therapies while deepening our understanding of kidney diseases.

The regulation of OATs involves multiple levels, including transcriptional, post-transcriptional, and post-translational regulations. Post-translational modification is the covalent modification of a target protein (e.g., OATs) after it has been translated and released from the ribosome [2,3,7,8]. Palmitoylation is an essential post-translational modification that is involved in modulating protein expression and functions. This process is the covalent attachment of palmitate molecules to cysteine residues on substrate proteins. Palmitoylation is a dynamic and reversible modification in cells [2,9,10]. Thus, the cycle of palmitoylation and depalmitoylation plays a significant role in the localization, trafficking, stability, and functions of those modified proteins. In mammalian cells, the palmitoylation process is mediated by palmitoyltransferases that catalyze the conjugation of palmitate molecules to substrate proteins [11,12,13,14]. In the context of membrane proteins, palmitoylation has only been investigated in a limited number of transporters [15]. Whether OATs are subject to the regulation by palmitoylation is completely unknown.

Epidermal growth factor (EGF) signaling is essential for many fundamental cellular processes, such as cell proliferation, migration, survival, and differentiation. Depending on the tissue, EGF signaling is also involved in the initiation and progression of numerous diseases [16,17,18,19]. Human kidneys are a major producer of EGF, along with skeletal muscle and the pancreas. EGF exerts its biological functions via binding to the EGF receptor (EGFR) on the cell surface, which leads to the activation of the intracellular tyrosine kinase domain of EGFR. Upon activation, EGFR could activate various downstream signaling molecules, including extracellular signal-regulated kinase (ERK) and protein kinase B (PKB) [18]. EGF signaling plays diverse roles in renal physiology and pathology in human kidneys, such as tubulogenesis and tubular regeneration after renal injury [18,19,20]. EGF and its receptor could control renal sodium reabsorption by regulating the epithelial sodium channel [16,21]. EGFR also helps maintain magnesium homeostasis in the renal distal tubules via modulating one of the magnesium channels [16,22]. EGF signaling modulates the osmotic water permeability in the renal collecting duct by regulating the aquaporin-2 water channel (AQP2) [23].

In the current study, we investigated the regulatory effects of EGF signaling on OAT3. Specifically, we focused on the effects of the signaling pathway on the palmitoylation status, protein expression, and ultimately, the transport activity of OAT3.

## 2. Materials and Methods

### 2.1. Materials

Parental COS-7 and HEK-293 cells were obtained from ATCC (Manassas, VA, USA). [^3^H]-labeled estrone sulfate was purchased from PerkinElmer (Waltham, MA, USA). Membrane-impermeable biotinylation reagent sulfo-NHS-SS-biotin and streptavidin agarose resin were purchased from Thermo Scientific (Rockford, IL, USA). Mouse anti-myc antibody (9E10) was purchased from Roche (Indianapolis, IN, USA). Mouse anti-E-cadherin antibody was purchased from Abcam (Cambridge, MA, USA). Mouse anti-GAPDH antibodies were purchased from Santa Cruz Biotechnology (Dallas, TX, USA). Biotin-azide and 17-Octadecynoic acid (17-ODYA) were purchased from Cayman Chemical (Ann Arbor, MI, USA). Activated Thiol-Sepharose 4B, Tris[(1-benzyl-1H-1,2,3-triazol-4-yl)methyl]amine (TBTA), Tris(2-carboxyethyl)phosphine hydrochloride (TCEP) solution, Dibutyryl cyclic-AMP sodium salt (Bt2-cAMP), H-89 dihydrochloride hydrate (H-89), 2-Bromohexadecanoic acid (2-BP), epidermal growth factor (EGF), and all other reagents were purchased from Sigma-Aldrich (St. Louis, MO, USA).

### 2.2. Cell Culture and Transfection

Parental green monkey kidney COS-7 cells were cultured in Dulbecco’s modified Eagle’s medium (DMEM) (Invitrogen, Carlsbad, CA, USA) containing 10% fetal bovine serum (FBS) (Thermo Fisher Scientific, Waltham, MA, USA) in a standard cell culture incubator that maintains 37 °C and 5% CO_2_ conditions. As previously reported, COS-7 cells stably expressing myc-tagged human OAT3 were established in our laboratory [24], which were grown in a standard DMEM medium containing 10% FBS and 0.5 mg/mL G418 antibiotic.

### 2.3. Transport Activity Measurement (Uptake Assay)

The uptake assay of OAT3-expressing COS-7 cells was performed following our laboratory’s previously established protocol [24,25]. Briefly, cells were grown in 48-well culture plates for 24 h before performing the assay. A 4 min uptake study was performed with the uptake solution that contained phosphate-buffered saline (PBS) and 300 nM [^3^H]-estrone sulfate (ES) at room temperature. The uptake process was stopped by removing the uptake solution and washing it with cold PBS. Then, the cells were lysed and mixed with a biodegradable scintillation cocktail for further analysis using HIDEX 300 SL (HIDEX, Turku, Finland). The transport activity values, which were calculated as a percentage compared to control groups, reveal the changes in the OAT3 transport function.

### 2.4. Biotinylation Assay

The assessment of OAT3 protein expression at the cell membrane was conducted following our laboratory’s previously established protocol [24,25]. Briefly, cell surface proteins were labeled with membrane-impermeable biotinylation reagent Sulfo-NHS-SS-biotin (0.5 mg/mL in PBS pH 8.0) on ice, followed by quenching the unreacted reagent with 100 mM glycine in cold PBS. Cell lysate was incubated overnight at 4 °C with streptavidin resin to isolate biotin-labeled surface proteins. Then, cell surface OAT3 was analyzed by SDS-PAGE and immunoblotting using an anti-myc antibody. The epitope myc was tagged on OAT3 for immune detection of the transporter.

### 2.5. Click Chemistry-Based Labeling Assay

The method for detection of palmitoylated OAT3 protein was based on a previously published article with modifications [26]. In brief, cells were labeled with 17-ODYA, which selectively labeled newly palmitoylated proteins. The cell lysates were centrifuged at 16,000× *g* at 4 °C. Around 100 µg of supernatants were combined with 1 mM TCEP, 100 µM TBTA, 300 µM biotin-azide, and 1 mM of CuSO_4_ for a click chemistry reaction at room temperature for 1 h. During the reaction, 17-ODYA-labeled proteins were reacted with biotin-azide and transformed into biotin-labeled proteins. The mixtures were then transferred to prewashed streptavidin resin to separate these biotin-labeled proteins. After overnight incubation at 4 °C, SDS-PAGE and immunoblotting with anti-myc antibody were used to identify palmitoylated OAT3 protein. OAT3 expression in whole-cell lysates before the click reaction was also measured through SDS-PAGE and immunoblotting as the total loading control.

### 2.6. Resin-Assisted Capture (RAC) Assay

The RAC method for detection of palmitoylated protein was previously published and modified by our laboratory [27,28]. Briefly, COS-7 cells were lysed. The cell lysate was centrifuged at 16,000× *g* at 4 °C for 20 min, and the supernatants were incubated in blocking buffer (100 mM HEPES, 1.0 mM EDTA, 2.5% SDS, 0.1% MMTS in dH_2_O pH 7.5) at 42 °C. MMTS is a sulfhydryl-reactive compound, which blocks free thiol groups. Then, four volumes of −20 °C acetone were added to precipitate proteins at −20 °C overnight. The mixture was then centrifuged at 16,000× *g* at 4 °C. The pellets were then washed with −20 °C 80% acetone (16,000× *g*, 1 min), air-dried, and resuspended in binding buffer (100mM HEPES, 1.0 mM EDTA, 1% SDS in dH_2_O pH 7.5). Neutral hydroxylamine was added to the mixture to cleave thioester bonds and expose the thiol groups. Palmitoylated proteins were then captured by Activated Thiol-Sepharose 4B resin. After binding at room temperature with resins for 3 h, palmitoylated OAT3 expression was measured through SDS-PAGE and immunoblotting with an anti-myc antibody. Whole-cell lysates before resin binding were collected, and OAT3 was detected with an anti-myc antibody as the total loading control.

### 2.7. SDS-PAGE and Immunoblotting

Briefly, denatured proteins were separated on 7.5% SDS-PAGE mini-gels (Bio-Rad, Hercules, CA, USA) and were transferred onto polyvinylidene difluoride (PVDF) membranes. The membranes were incubated in 5% nonfat milk in PBST buffer (0.1% Tween-20 in PBS) at room temperature for 3 h to block undesired signal and then incubated with the indicated primary antibody at 4 °C overnight. After washing with PBST to remove the excess primary antibodies, the blots were incubated with horseradish peroxidase-conjugated secondary antibodies. SuperSignal West Dura Extended Duration Substrate kit (Pierce Biotechnology, Rockford, IL, USA) and ChemiDoc Imaging System (Bio-Rad, Hercules, CA, USA) were used to detect target proteins’ signals. The densities of non-saturating, immunoreactive protein bands of interest were quantified using Imaging Lab 6.0 software (Bio-Rad, Hercules, CA, USA).

### 2.8. Data Analysis

Each experiment was conducted independently at least three times, with multiple replicates for statistical analysis. Student’s paired *t*-tests were applied for comparisons between two groups, while one-way ANOVA followed by Tukey’s post hoc test was used for multiple-group comparisons. Statistical analyses were performed using GraphPad Prism 9.5 software (GraphPad Software, San Diego, CA, USA), with a *p* value of <0.05 considered statistically significant.

## 3. Results

### 3.1. Effect of EGF on OAT3 Transport Activity

**Figure 1:**
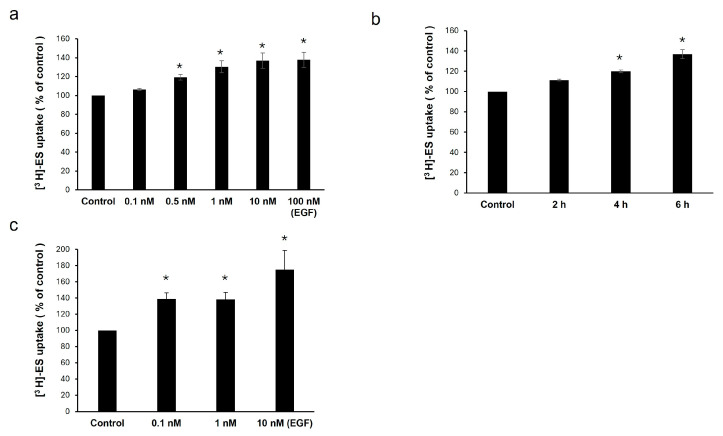
Effect of EGF on OAT3 transport activity. (**a**) OAT3-expressing COS-7 cells were treated with EGF for 6 h at different concentrations. The uptake of [^3^H]-estrone sulfate (ES) for 4 min was then performed. (**b**) OAT3-expressing COS-7 cells were treated with 1nM EGF for different time periods. The uptake of [^3^H]-estrone sulfate (ES) for 4 min was then performed. (**c**) OAT3-expressing HEK293 cells were treated with EGF for 6 h at different concentrations. Followed by 4 min [^3^H]-estrone sulfate (ES) uptake. Uptake activity was expressed as the percentage of uptake measured in the control group. Three independent experiments were performed to collect data. Values are means ± S.D. (n = 3). * *p* < 0.05.

To explore the effect of EGF on OAT3 activity, we treated COS-7 cells stably expressing OAT3 with different concentrations of EGF (0.1–100 nM) and different time periods (2–6 h), followed by measuring the uptake of OAT3-mediated [^3^H]-estrone sulfate (ES) into the cells. Estrone sulfate is an OAT3-specific substrate. EGF-stimulated OAT3-mediated transport in a dose-dependent manner with maximum stimulation of ~35–40% at 1–100 nM EGF (Figure 1a). The stimulation of OAT3-mediated transport by EGF also increased with treatment time, with ~40% stimulation at 6 h (Figure 1b). Uptake assays in OAT3-expressing HEK293 cells derived from human kidneys also showed a similar stimulatory effect from EGF treatment (Figure 1c).

### 3.2. The Role of Protein Kinase A (PKA) in the Effect of EGF on OAT3 Transport Activity

**Figure 2:**
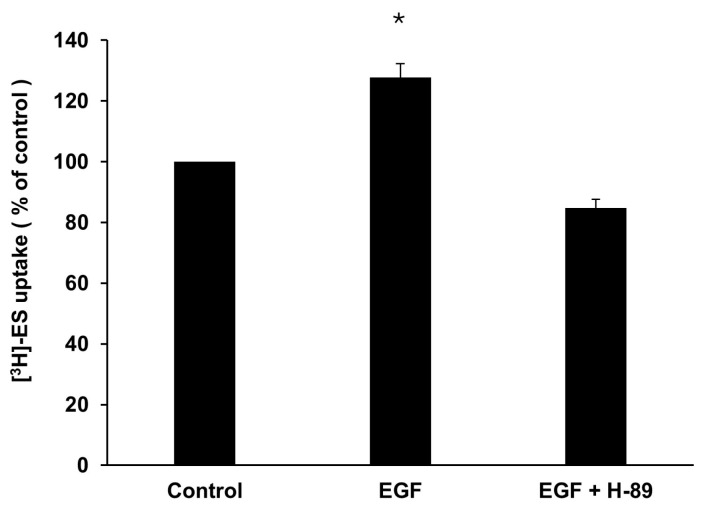
The role of protein kinase A (PKA) in the effect of EGF on OAT3 transport activity. OAT3-expressing COS-7 cells were treated with 1 nM EGF for 6 h with or without 20 µM H-89, a PKA inhibitor. A 4 min uptake of [^3^H]-estrone sulfate (ES) was then performed. Three independent experiments were performed, and the uptake value in each group is expressed as a percentage of the uptake value measured in control cells. Values are mean ± S.D. (n = 3). * *p* < 0.05.

To determine if EGF-stimulated OAT3 transport activity through the PKA pathway, we treated OAT3-expressing cells with 1nM EGF in the presence or absence of H-89, a PKA inhibitor, followed by measuring OAT3-mediated uptake of [^3^H]-estrone sulfate (ES) into the cells. As shown in Figure 2, treatment with EGF resulted in a significant increase in OAT3 transport activity, and such an increase was blocked in the presence of H-89.

### 3.3. Effect of EGF on OAT3 Expression

**Figure 3:**
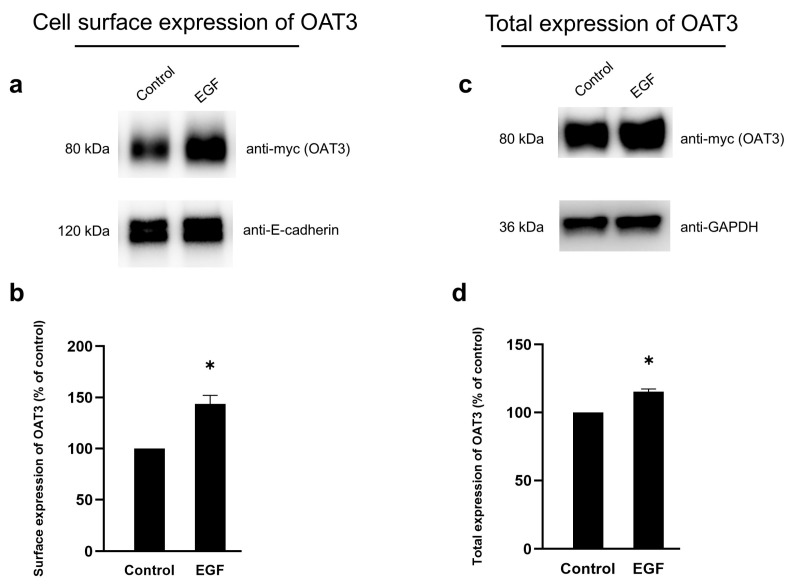
Effect of EGF on OAT3 expression. (**a**) Top panel: The effect on cell surface expression of OAT3. OAT3-expressing cells were treated with EGF (1 nM, 6 h). The cell surface expression of OAT3 was then determined by a biotinylation method in conjunction with immunoblotting with anti-myc antibody. Epitope myc was tagged to OAT3 for immunodetection. Bottom panel: The same blot was re-probed with anti-E-cadherin, which is a cell membrane protein marker. (**b**) The densitometry of Figure 3a and other repeats. Density values were normalized to control protein E-cadherin. Values are mean ± S.D. (n = 3). * *p* < 0.05. (**c**) Top panel: The effect of EGF on total expression of OAT3. After treatment with EGF (1 nM, 6 h), the OAT3-expressing cells were lysed, followed by immunoblotting, and OAT3 was detected with anti-myc antibody. Bottom panel: The same blot was re-probed with anti-GAPDH. GAPDH is a cellular protein marker. (**d**) The densitometry of Figure 3c and other repeats. Density values were normalized to control protein GAPDH. Values are mean ± S.D. (n = 3). * *p* < 0.05.

To examine the effect of EGF on OAT3 expression, we treated OAT3-expressing cells with 1 nM EGF, followed by measuring OAT3 expression using a biotinylation strategy, as described in the Section 2. Our results showed that EGF stimulated OAT3 expression both at the cell surface (Figure 3a, top panel) and in total cell lysate (Figure 3c, top panel). E-cadherin, a cell surface protein marker (Figure 3a, bottom panel), and anti-GAPDH, a cellular protein marker (Figure 3c, bottom panel), were used as loading controls.

### 3.4. Effect of EGF on OAT3 Palmitoylation

**Figure 4:**
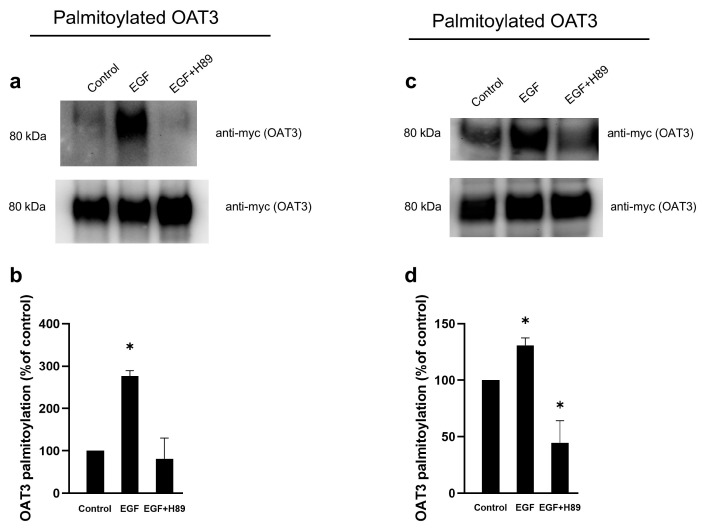
The effect of EGF on OAT3 palmitoylation. (**a**) Top panel: OAT3 palmitoylation was measured by click chemistry-based labeling, as described in the Section 2. OAT3-expressing COS-7 cells were incubated with 1nM EGF for 6 h, with or without 20 µM H-89, a PKA inhibitor, followed by click chemistry-based labeling in conjunction with immunoblotting with anti-myc antibody. Bottom panel: OAT3 was detected from total-cell lysates as the loading control for each sample. (**b**) The densitometry of Figure 4a, top panel, and other repeats. Density values were normalized to OAT3 in total-cell lysates. Values are mean ± S.D. (n = 3). * *p* < 0.05. (**c**) Top panel: OAT3 palmitoylation measured by resin-assisted capture assay. After treating OAT3-expressing COS-7 cells with EGF (1 nM, 6 h) and with or without H-89 (20 µM, 6 h), the palmitoylated OAT3 was determined by resin-assisted capture assay, in conjunction with immunoblotting with an anti-myc antibody. Bottom panel: OAT3 was detected from total-cell lysates as the loading control. (**d**) The densitometry of Figure 4c and other repeats. Density values were normalized to OAT3 in total-cell lysates. Values are mean ± S.D. (n = 3). * *p* < 0.05.

The relationship between EGF and palmitoylation was examined by two independent methods. First, we employed a click chemistry-based labeling approach, as described in the Section 2. EGF significantly enhanced OAT3 palmitoylation, and such enhancement was blocked in the presence of H-89, a PKA inhibitor (Figure 4a, top panel). Figure 4a, bottom panel, shows OAT3 expression in total cell lysate, used as a loading control. The above result was then independently confirmed by another method called resin-assisted capture assay, as described in the Section 2 (Figure 4c, top panel). Figure 4c, bottom panel, shows OAT3 expression in total cell lysate before resin-assisted capture, used as a loading control.

### 3.5. Effect of 2-BP on OAT3 Palmitoylations

**Figure 5:**
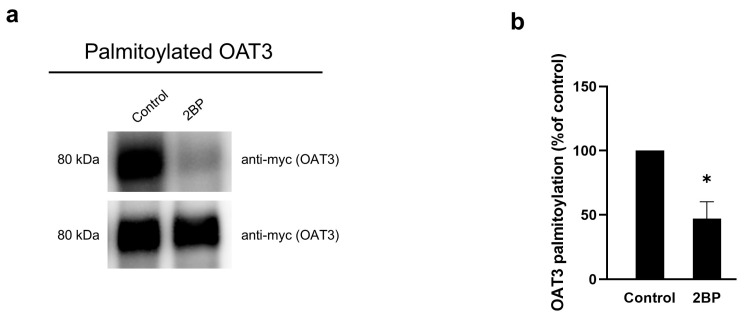
Effect of 2-BP on OAT3 palmitoylation. (**a**) Top panel: OAT3-expressing cells were treated with 2-BP at 10 µM for 4 h. OAT3 palmitoylation was examined by a click chemistry-based labeling method as described in the Section 2. Palmitoylated OAT3 was detected by immunoblotting with anti-myc antibody. Bottom panel: OAT3 was detected from total-cell lysates as the loading control for each sample. (**b**) The densitometry of Figure 5a and other repeats. Density values were normalized to OAT3 in total-cell lysates. Values are mean ± S.D. (n = 3). * *p* < 0.05.

Our results above showed that EGF-stimulated OAT3 transport activity, expression, and palmitoylation through the PKA pathway. Palmitoylation is catalyzed by a group of palmitoyltransferrases, and 2-BP is a general inhibitor for palmitoyltransfereases. Here, we examined the effect of 2-BP on OAT3 palmitoylation by a click chemistry-based labeling approach, as described in the Section 2. We observed a significant decrease in OAT3 palmitoylation when cells were treated with 2-BP at 10 µM for 4 h. (Figure 5a, top panel). Figure 5a, bottom panel, shows OAT3 expression in total cell lysate, used as a loading control.

### 3.6. Effect of 2-BP on OAT3 Expression

**Figure 6:**
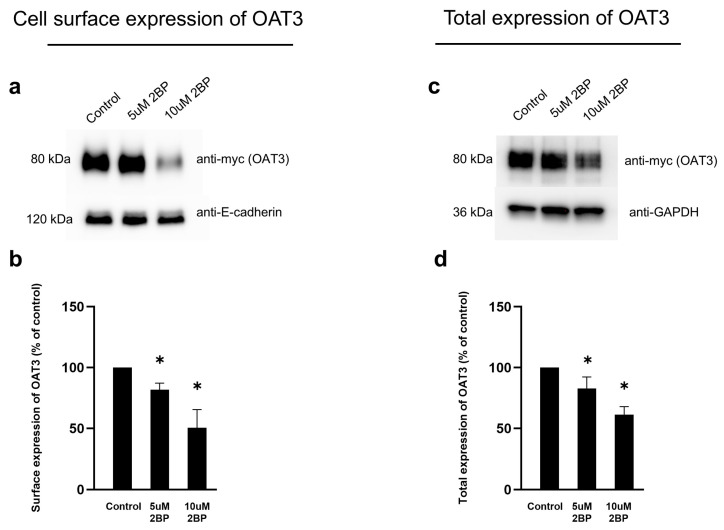
The effect of 2-BP on OAT3 expression. (**a**) Top panel: The effect of 2-BP on OAT3 expression at the cell surface. OAT3-expressing cells were treated with 2-BP (5 µM or 10 µM, 4 h). OAT3 expression at the cell surface was examined by the biotinylation method in conjunction with immunoblotting with anti-my antibody. Bottom panel: The same blot was re-probed with anti-E-cadherin, which is a cell membrane protein marker, as a loading control. (**b**) The densitometry of Figure 6a and other repeats. Density values were normalized to control protein E-cadherin. Values are mean ± S.D. (n = 3). * *p* < 0.05. (**c**) Top panel: The effect of 2-BP on OAT3 expression in total cell lysate. After treatment with 2-BP (5 µM or 10 µM, 4 h), the OAT3-expressing cells were lysed, followed by immunoblotting with anti-myc antibody. Bottom panel: The same blot was re-probed with anti-GAPDH. GAPDH is a cellular protein marker as a loading control. (**d**) The densitometry of Figure 6c and other repeats. Density values were normalized to control protein GAPDH. Values are mean ± S.D. (n = 3). * *p* < 0.05.

The effect of 2-BP on OAT3 expression on the cell surface and in total cell lysate was examined using a biotinylation method described in the Section 2, as shown in Figure 6. Although there were no significant changes in the cell surface expression and total expression of OAT3 between the control and 5 µM 2-BP, a clear decrease in OAT3 expression was observed at 10 µM 2-BP (Figure 6a, top panel, and Figure 6c, top panel). E-cadherin, a cell surface protein marker (Figure 6a, bottom panel), and anti-GAPDH, a cellular protein marker (Figure 6c, bottom panel), were used as loading controls.

### 3.7. The Role of Palmitoylation in EGF/PKA Stimulation of OAT3 Transport Activity

**Figure 7:**
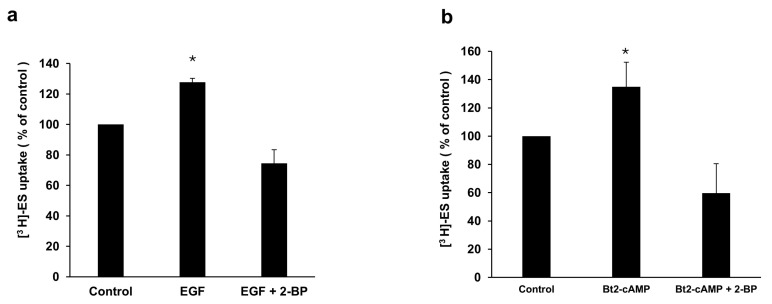
The role of palmitoylation in EGF/PKA stimulation of OAT3 transport activity. (**a**) OAT3-expressing COS-7 cells were treated with 1nM of EGF, with or without 1 µM of 2-BP, for 6 h, followed by measuring the uptake of [^3^H]-estrone sulfate (ES) into the cells. (**b**) OAT3-expressing COS-7 cells were treated with 10 µM of Bt2-cAMP, a PKA activator, with or without 1 µM of 2-BP for 4 h, followed by measuring the uptake of [^3^H]-estrone sulfate (ES) into the cells. Uptake activity was expressed as the percentage of uptake measured in the control group. Three independent experiments were performed to collect data. Values are means ± S.D. (n = 3). * *p* < 0.05.

To examine the role of palmitoylation in EGF/PKA stimulation of OAT3 transport activity, we treated OAT3-expressing cells with EGF in the presence and absence of 2-BP. The OAT3 transporter activity was then examined by measuring the uptake of [^3^H]-estrone sulfate (ES) into the cells. Our result showed that EGF induced a significant upregulation of OAT3 transport activity, and such upregulation was blocked by 2-BP (Figure 7a). Similarly, we also treated OAT3-expressing cells with Bt2-cAMP, a PKA activator, in the presence and absence of 2-BP, followed by the measurement of OAT3 transporter activity. Our result showed that Bt2-cAMP induced a significant upregulation of OAT3 transport activity, and such upregulation was blocked by 2-BP (Figure 7b).

### 3.8. Effect of EGF and Osimertinib on OAT3 Transport Activity

**Figure 8:**
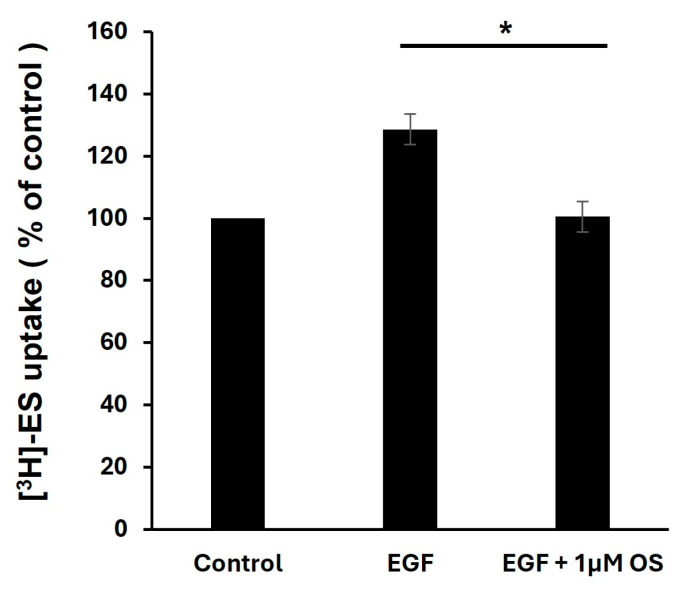
The effect of EGF and osimertinib (OS) on OAT3 transport activity. OAT3-expressing COS7 cells were treated with 1 nM EGF with or without OS (1 µM) for 6 h. Followed by 4 min [^3^H]-estrone sulfate (ES) uptake. Three independent experiments were performed, and the uptake value in each group is expressed as a percentage of the uptake value of control cells. Values are mean ± S.D. (n = 3). * *p* < 0.05.

Osimertinib is one of the FDA-approved anti-cancer therapies/EGFR inhibitors [29,30]. Therefore, we tested its effect on EGF regulation of OAT3 transporter activity. Our data showed that 1 µM osimertinib significantly blocked the EGF-stimulated OAT3 activity (Figure 8).

## 4. Discussion

OAT3, predominantly expressed in the kidney, is responsible for the active transport of various anionic substrates from blood circulation into the renal proximal tubules for subsequent elimination in the urine. OAT3 substrates are diverse, including endogenous molecules, metabolites, and exogenous medications. Clinically important drug substrates of OAT3 include antibiotics, NSAIDs, methotrexate, antivirals, and HIV protease inhibitors [4,31]. Impaired OAT3 function could result in the reduced or delayed renal elimination of OAT3 substrate drugs. Furthermore, because of the ability of OAT3 to interact with a wide range of drugs, drug–drug interactions can occur at the transporter molecule, causing unwanted changes in the pharmacokinetic and toxicological profile of these drugs [2,3,4,6]. The regulatory guidelines from the US Food and Drug Administration (FDA) and European Medicines Agency (EMA) recommend evaluating the potential of OAT3-mediated drug–drug interactions for new drug candidates and their important metabolites, particularly where the negatively charged drugs and their primary metabolites are eliminated through the kidney in a significant amount [6,32]. Therefore, investigating the regulatory mechanism controlling OAT3 transport function and expression is of great importance in drug development.

In the current study, we revealed the regulatory effects of the EGF/PKA signaling pathway on OAT3 expression and transport activity. Importantly, our study demonstrated that palmitoylation of OAT3, a novel type of post-translational modification, played a pivotal role in mediating the effects of EGF/PKA signaling on OAT3. Our study also provided an important implication that during comorbidity therapies, anti-EGFR inhibitors/drugs such as osimertinib could potentially decrease the transport activity of renal OAT3, which would subsequently alter the therapeutic efficacy and toxicity of many co-medications that are OAT3 substrates.

Our experimental results showed that EGF induced time- and concentration-dependent stimulation of OAT3 transport activity, reaching a maximum stimulation of ~30–40% (Figure 1). This stimulation of OAT3 transport activity by EGF was abrogated by H-89 (Figure 2), a PKA inhibitor, indicating that PKA is the downstream signaling molecule of the EGF effect. Our results (Figure 3) further showed that EGF significantly increased OAT3 expression at the cell surface and in total cell lysate, supporting that EGF-enhanced OAT3 transport activity was largely due to EGF-enhanced OAT3 expression. The physiological concentration of EGF in human plasma is about 0.02–0.3 nM, and 1–30 nM of EGF has often been used in many in vitro studies to examine the biological effects of EGF [17,33,34,35]. Thus, to study the effects of EGF on OAT3, the EGF concentration (1 nM) chosen in the current study is appropriate and physiologically relevant.

According to previous studies, EGF reduces the palmitoylation of CUB domain-containing protein 1 (CDCP1) and inhibits the palmitoylation-dependent degradation of CDCP1 to promote its expression on the cell surface of ovarian cancer cells [36,37]. To explore the potential relationship between EGF/PKA signaling and the palmitoylation of OAT3, we employed two independent approaches: click chemistry-based labeling and resin-assisted capture (RAC). Both approaches yielded consistent results that EGF-stimulated OAT3 palmitoylation and such stimulation was blocked by H-89, a PKA inhibitor (Figure 4), suggesting that activation of the EGF/PKA signaling pathway promotes OAT3 palmitoylation. OAT3 palmitoylation is a novel type of post-translational modification in the OAT field. Palmitoylation is catalyzed by a group of palmitoyltransferases, and 2-BP is a general inhibitor for palmitoyltransferases. Treatment of OAT3-expressing cells with 2-BP indeed led to a significant decrease in OAT3 palmitoylation (Figure 5). We further showed that treatment of OAT3-expressing cells with 2-BP resulted in a dose-dependent decrease in OAT3 expression (Figure 6). Most importantly, we showed that 2-BP was able to abrogate the stimulating effects from EGF and Bt2-cAMP (PKA activator) on OAT3 activity (Figure 7), confirming the EGF/PKA effects on OAT3 were via altering the palmitoylation of OAT3.

Protein palmitoylation plays an essential role in modulating protein expression, localization, trafficking, stability, and functions [13,38]. Through controlling its target proteins and signaling pathways, palmitoylation is involved in numerous human diseases [11,12]. For instance, the palmitoylation of angiotensin-converting enzyme 2 (ACE2) was critical for its targeting to the cell membrane and following secretion via extracellular vesicles. Such ACE2-enhanced extracellular vesicles exhibited a higher affinity to the SARS-CoV-2 virus and were able to protect mice from the SARS-CoV-2-induced lung infection [39]. Polycystin 1 (PC1) was proven to be palmitoylated at its carboxyl terminal, and this modification was critical for the expression and localization of the PC1 protein, which contributed to the progression of autosomal dominant polycystic kidney disease [40]. Protein palmitoylation was down-regulated in the mouse model of kidney fibrosis as well as in chronic kidney disease patients. Mechanistically, the palmitoylation of β-catenin modulated its degradation and expression level, which was considered a promoting factor in kidney fibrosis [41]. As a representative oncogene, the palmitoylation of NRAS was shown to control its cellular localization and the activation of downstream signaling pathways that often lead to oncogenic cell transformations. The mice transplanted with bone marrow cells expressing oncogenic human NRAS protein developed a leukemia-like disease and died within a few months, while the mice transplanted with cells expressing palmitoylation mutant of NRAS did not develop such a disease and stayed healthy [12,42].

We showed that 1 µM osimertinib, an FDA-approved anti-cancer drug/EGFR inhibitor, significantly blocked the EGF-stimulated OAT3 activity (Figure 8). This finding has significant implications for clinical drug therapies. EGF exerts its effects through binding to the EGF receptor (EGFR), and its signaling pathways play essential roles in cancer development and anti-cancer therapies. For example, lung cancer is one of the leading causes of cancer-related mortality, and non-small cell lung cancer (NSCLC) accounts for about 90% of all lung cancer [43]. Many NSCLC patients carry activating mutations of EGFR. These activating mutations of EGFR would lead to constitutively active EGFR signaling, which often leads to cellular transformations and carcinogenesis [29,44,45]. EGFR mutations contributing to NSCLC usually occur in the tyrosine kinase domain of EGFR, which has been targeted as a therapeutic approach via the successful development of anti-EGFR tyrosine kinase inhibitors [29]. Currently, osimertinib is the first-line therapy against advanced EGFR-mutant NSCLC. Osimertinib holds high inhibitory efficacy against many EGFR mutations in patients with increased specificity and reduced toxicity [29,30,46,47].

Remote sensing and signaling is a novel theory demonstrating the complex regulation network among multiple tissues and organs to maintain homeostasis throughout the human body. The regulation of transporters from distal signaling molecules is indeed one of the critical points of this network. Endogenous signaling molecules such as hormones are released by one organ and reach other tissues through blood circulation to regulate transporters there [1,48,49,50,51,52,53]. In the current study, EGF is a good example of remote regulation: it is a growth factor synthesized and released by multiple tissues. Through system circulation, EGF binds to EGFR expressed in the kidney and is able to regulate the expression and transport activity of renal OAT3.

## 5. Conclusions

**Figure 9:**
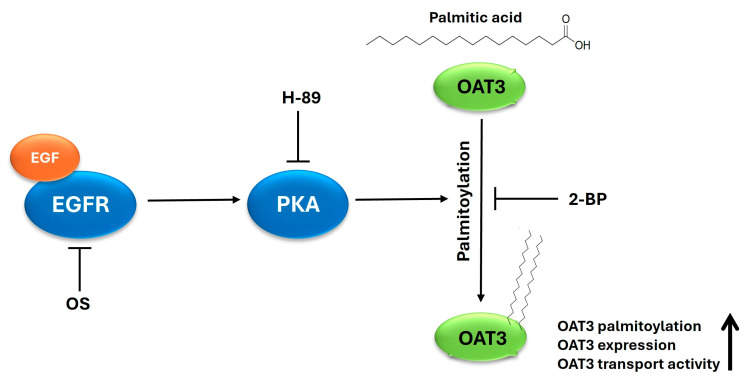
Regulation of OAT3 by palmitoylation and EGF/PKA signaling. EGF, epidermal growth factor. EGFR, epidermal growth factor receptor. OS, osimertinib. PKA, protein kinase A. OAT3, organic anion transporter 3. 2-BP, 2-bromopalmitic acid.

In conclusion, our current investigation demonstrated that (I) EGF-regulated OAT3 function occurs through the EGF/PKA signaling pathway, (II) palmitoylation mediates the effect of EGF/PKA on OAT3, and (III) an FDA-approved anti-cancer drug/EGFR inhibitor, osimertinib, inhibits OAT3 activity, providing the important implication that during comorbidity therapies, osimertinib and other anti-cancer drugs/EGFR inhibitors could potentially decrease the transport activity of renal OAT3, which would subsequently alter the therapeutic efficacy and toxicity of many co-medications that are OAT3 substrates (Figure 9).

## Data Availability

The original contributions presented in this study are included in the article; further inquiries can be directed to the corresponding author.
